# Specific or not specific recruitment of DNMTs for DNA methylation, an epigenetic dilemma

**DOI:** 10.1186/s13148-018-0450-y

**Published:** 2018-02-09

**Authors:** Eric Hervouet, Paul Peixoto, Régis Delage-Mourroux, Michaël Boyer-Guittaut, Pierre-François Cartron

**Affiliations:** 1 INSERM unit 1098, University of Bourgogne Franche-Comté, Besançon, France; 2 EPIGENExp (EPIgenetics and GENe EXPression Technical Platform), Besançon, France; 3 INSERM unit S1232, University of Nantes, Nantes, France; 4 Institut de cancérologie de l’Ouest, Nantes, France; 5 REpiCGO (Cancéropole Grand-Ouest), Nantes, France; 6 EpiSAVMEN Networks, Nantes, Région Pays de la Loire France

**Keywords:** DNA methylation, DNMT1, DNMT3A, DNMT3B, DNMT3L, Epigenetics, DNMT-including complexes

## Abstract

Our current view of DNA methylation processes is strongly moving: First, even if it was generally admitted that DNMT3A and DNMT3B are associated with de novo methylation and DNMT1 is associated with inheritance DNA methylation, these distinctions are now not so clear. Secondly, since one decade, many partners of DNMTs have been involved in both the regulation of DNA methylation activity and DNMT recruitment on DNA. The high diversity of interactions and the combination of these interactions let us to subclass the different DNMT-including complexes. For example, the DNMT3L/DNMT3A complex is mainly related to de novo DNA methylation in embryonic states, whereas the DNMT1/PCNA/UHRF1 complex is required for maintaining global DNA methylation following DNA replication. On the opposite to these unspecific DNA methylation machineries (no preferential DNA sequence), some recently identified DNMT-including complexes are recruited on specific DNA sequences. The coexistence of both types of DNA methylation (un/specific) suggests a close cooperation and an orchestration between these systems to maintain genome and epigenome integrities. Deregulation of these systems can lead to pathologic disorders.

## Background

### DNA methyl transferases are the catalytic players of DNA methylation

DNA methylation, occurring in CpGs motifs, is the reaction catalyzing the covalent transfer of a methyl group from S-andenosyl methionine (SAM) to the fifth carbon of cytosines (C). DNA methylation is involved in numerous biological events (e.g., embryonic development, parental imprinting genes, transposon silencing, X inactivation, cancer), and it concerns about 70–80% of CpGs in mammalian DNA. DNA methylation, which is generally observed in a condensed chromatin and associates with transcriptional gene silencing when it occurs in promoters, is processed by two distinct mechanisms: (i) the inheritance DNA methylation that allows the maintenance of DNA methylation marks on the new strand using the parental methylated strand as a matrix, following DNA replication and (ii) de novo DNA methylation which occurs on both strands independently of DNA replication. De novo methylation happens predominantly during embryogenesis and is further maintained by the DNA methylation inheritance machinery after DNA replication and cell division. DNA methylation is processed by a family of enzymes, the DNA methyl transferases (DNMTs), which are divided in three classes: DNMT1, DNMT2, and the DNMT3A/3B/3L. The DNMT2 functions have been poorly investigated: This enzyme may methylate the consensus sequence TTNCGGAR but DNMT2 is probably mainly involved in the methylation of C38 of tRNA^Asp^ [1, 2]. Our review will focus on DNMT1, DNMT3A, DNMT3B, and DNMT3L.

### DNMT1, the major enzyme involved in DNA methylation inheritance

**Fig. 1:**
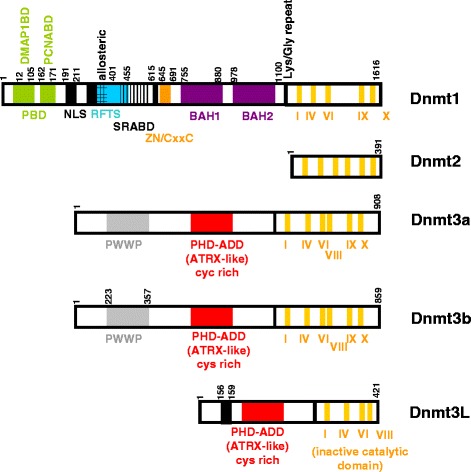
Representative structure of DNMTs. Adapted from [1–8]

DNMT1, a large protein of 1616 amino acids (aa) which mainly catalyzes DNA methylation inheritance activity, is composed of a large regulator N-terminal region (1000 aa) and a small catalytic C-terminal region. In this last region, 10 catalytic domains are essential for the interaction with the SAM. C-ter and N-ter regions are linked by 12 repeats of KG di-peptides. The N-ter region is composed of (i) a binding protein domain able to interact with a large panel of proteins [3], (ii) a RFTS (replication focus targeting sequence) domain involved in the recruitment of DNMT1 into the DNA replication fork, (iii) a zinc-binding domain, (iv) some BAH domains (adjacent homology domain), and (v) a nuclear localization signal (NLS) (aa 191–211) (Fig. 1). However, the role of the N-ter region in DNMT1 activity remains unclear. Some authors showed that DNMT1 activity was independent from this region [4–6]. Others reported that the interaction of the N-ter region with the C-ter region, which is promoted by the S515 phosphorylation, was required for tri-dimensional (3D) modification of DNMT1 and its activity. Furthermore, DNMT1 also presents an allosteric site (aa 284–287: independent from the catalytic site) which may bind 5mC and increase fit for both SAM and DNA [7].

### DNMT3A and DNMT3B, the enzymes predominantly associated with de novo DNA methylation

De novo DNA methylation activity, catalyzed by DNMT3A and DNMT3B, is essential during embryonic development or gametogenesis but is also frequently associated to aberrant gene repression in many pathologies (e.g., cancer) [8]. The structures of DNMT3A and DNMT3B are very close and are composed of (i) a N-terminal region comprising a PWWP domain which is essential for DNA binding, (ii) a PHD-like ADD domain involved in protein/protein interactions, and (iii) a C-terminal region responsible for the catalytic activity (Fig. 1). A small preference for the recruitment on unmethylated DNA was seen for DNMT3A whereas DNMT3B might link both hemi and unmethylated DNA. Finally, a third but catalytically inactive member of the DNMT3 family, DNMT3L, which is mainly expressed during development, is required for gene imprinting and the regulation of DNMT3A/B.

**Fig. 2:**
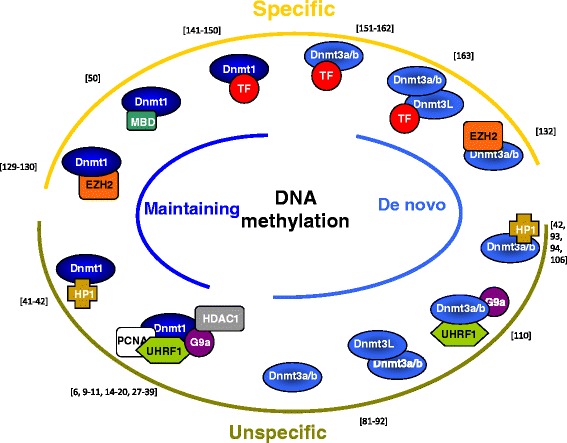
Schema of the main kinds of DNMT-including complexes. Maintaining (mainly catalyzed by DNMT1: dark blue curve) or de novo (mainly catalyzed by DNMT3A and DNMT3B: light blue curve) DNA methylation can be processed by numerous DNMT-including complexes which are either not specific (green curve) or specific (yellow curve) of particular DNA sequences. Specific complexes included polycomb proteins or transcriptional factor (TF), whereas unspecific complexes included heterochromatin readers or replication associated proteins

Since 10 years, our knowledge on the roles of DNMTs in DNA methylation has highly raised. More and more partners of each DNMT have been reported, and our view of DNA methylation machineries is currently moving, let us understand that (i) crosstalks exist between de novo and maintaining DNA methylation machineries and (ii) DNA methylation can be mediated by different DNMT-including complexes and some of them are not associated with specific DNA sequences while other complexes may target the methylation in a specific loci. The different kinds of DNMT-including complexes involved in DNA methylation are summarized in Fig. 2 and will be discussed below.

## Unspecific DNA methylation machinery

### Unspecific DNA methylation inheritance

#### PCNA/UHRF/DNMT1/HDAC1/G9a complex

##### PCNA and UHRF1

Mutations in the RFTS domain of DNMT1 dramatically reduced its activity. Moreover, this RFTS domain is probably involved in the allosteric activation of DNMT1. Indeed, stable DNMT1 homodimers are linked by a hydrophobic interaction requiring the RFTS domain of each DNMT1. Although monomeric DNMT1 presented a 2- to 50-fold increase activity in the presence of hemi-methylated DNA, compared to methylated DNA, many different partners could also be involved in the recruitment of DNMT1 on hemi-methylated DNA [9–12]. A strong association between DNMT1 and the replication machinery may explain the large concomitance of DNA replication and maintaining of DNA methylation [13]. Numerous reports showed that DNMT1/PCNA (proliferative cell nuclear antigen) interaction was essential for DNMT1 activity [6, 14–18]. PCNA which bound to the new replicated DNA strand is assumed to take down from the DNA polymerase and consequently favor DNMT1 recruitment. Indeed, the DNMT1/PCNA interaction could also modify the structure of the RFTS DNMT1 domain and by a ricochet increase in both DNMT1 affinity for DNA and its activity [19]. Opposite studies showed that the disruption of the PCNA binding domain of DNMT1 (aa 51–255), only reduced from twofold the DNA methylation maintaining activity, suggesting that PCNA was not essential. Bostick et al. and Sharif et al. have reported that UHRF1/DNMT1 interaction (UHRF1, ubiquitin-like PHD and RING finger domain 1; also called ICBP90 (inverted CCAAT box binding protein of 90 kDa) in human or Np95 in mouse) was also involved in DNMT1 recruitment [20, 21]. UHRF1 was first described for its E3 ubiquitin ligase activity on histone H3. UHRF1 also promotes DNA methylation inheritance preferentially during mid to late S phase where it accumulates in nuclear loci and favors the targeting of DNMT1 on hemi-methylated DNA. The DNMT1/UHRF1 interaction involves the SRA (SET and RING) domain of UHRF1 which also recognizes hemi-methylated CpG and three independent regions of DNMT1 (aa 1–446; 401–615; 1081–1408) [22]. Crystal resolution of the SRA domain of UHRF1 and mutagenesis experiments revealed that R443, Y466, and D469 residues were essential for DNA binding [23–26]. Invalidation of PCNA/DNMT1/UHRF1 complex using competitor peptides of DNMT1/PCNA and DNMT1/UHFR1 interactions, or knock down of DNMT1, strongly decreased the global DNA methylation and induced severe defects such as mitotic catastrophe [27–32].

##### HDAC1 and G9a

The concomitant presence of several repressive marks has often been observed on silenced promoters. Indeed, the presence of 5mC is often correlated with histone deacetylation, suggesting that DNA methylation and histone regulation machineries thinly cooperate. Maintaining of DNA methylation in heterochromatin required DNMT1/HDAC1 interaction and the deacetylation of histones [33]. HDAC2 and the histone methyl transferase (HMT) G9a (which catalyzes the repressive marks mono-, di-, and less efficiently trimethylation of H3K9 and H3K27) could also be recruited in the DNMT1/UHRF1/HDAC1/HDAC2/G9a complex via direct DNMT1/G9a, UHRF1/G9a, and UHRF1/HDAC2 interactions. The role of G9a is still in debate, as on the one hand, its inhibition caused a DNA hypomethylation in some imprinting genes, but on the other hand, G9a was dispensable for maintaining DNA methylation in somatic cells [34, 35]. UHRF1 recruitment on repressed promoters is reinforced by the affinity of its tandem tudor domain with the N-ter tail of H3K9me2/3, independently of the DNA methylation status [36]. Nevertheless, overexpression of both UHRF1 mutants incapable of binding hemi-methylated DNA or incapable of binding H3K9me3 partially restored global DNA methylation [37]. In conclusion, the DNMT1/PCNA/UHRF1/HDAC/G9a complex is preferentially recruited on chromatin in S phase where it promotes DNA methylation, histone deacetylation, and H3K9/H3K27 methylation [38, 39].

#### Maintaining DNA methylation activity in other DNMT1-including complexes

Although most of the unspecific inheritance DNA methylation activity is probably processed by the DNMT1/PCNA/UHRF1/HDAC/G9a complex, additional DNMT1-including complexes have been reported.

##### Cooperation of DNMT1 with nucleosome-related proteins and HMTs (HP1, SUV39H1, SNF2H)

Following DNA replication, some nucleosomes are deposited on the new replicated strand. Kinetics of histone modifications and DNA methylation are highly related but are dependent of both, the local DNA sequence and the nature of DNMTs/HDACs/HMTs complexes recruited. Although, in vitro, the DNA methylation of heterochromatin can be catalyzed by free DNMT1 on mononucleosomes, the interaction of DNMT1 with the ATP-dependent nucleosome remodeler SNF2H (SNF2 homolog) strongly increased DNMT1 recruitment on these nucleosomes [40]. Some HMTs are predominantly associated with euchromatin such as G9a (found in the DNMT1/PCNA/UHRF1/HDAC/G9a, see above) while others such as SUV39H1 (suppressor of variegation 3–9 homolog 1; catalyzes H3K9me3) are recruited on heterochromatin. Both kinds of HMTs could also regulate the DNMT1 recruitment in a direct or indirect DNMT1/HMT interaction manner. Indeed, the recognition of H3K9me by HP1 (heterochromatin protein-1) may serve for a further recruitment of DNMT1/SUV39H1/HP1 complex [41, 42]. A similar mechanism has also been observed for the euchromatin G9a-mediated H3K9me2 methylation which could also be recognized by HP1. Indeed, G9a/HP1 interaction induced both an increase in G9a activity and DNMT1 recruitment [43]. Conversely, DNMT1 was required for H3 deacetylation and di- and trimethylation of H3K9 in cancer cells [44]. Similarly, DNA methylation and histone methylation need both DNMT1 and SUV39H in zebrafish [45, 46]. However, in some cases, the HMT/DNMT1 interaction could be independent from DNA methylation: For example, the DNMT1 mutant, depleted for its catalytic domain, was still able to control the H3K4 demethylase LSD1 (lysine-specific demethylase 1A, also called KDM1) recruitment and to induce gene repression without DNA methylation (e.g., *MAGEA10*) [47].

##### DNMT1/CFP1

CFP1 (CysxxCys finger protein 1), which presents a high affinity for unmethylated DNA, has been shown to interact with DNMT1 (via aa 169–493, TS and 970–1616) [48]. CFP1−/− ES cells, showed a reduction of 70% of DNA methylation in single copy genes while a specific inhibition of DNMT1/CFP1 interaction strongly decreased tumor growth of glioma cells in nude mice [49].

##### DNMT1/MBDs

MBDs (methyl-CpG-binding domain protein) are organized in a family of five proteins (MeCP2, MBD1–4) known to interact with methylated DNA and are also involved in the recruitment of DNMT1. Indeed, the MBD2/MBD3 heterodimer which preferentially interact with hemi-methylated DNA could also recruit DNMT1, during late S phase, in replication loci [50]. MeCP2 was also known to form a ternary repressor complex with HDAC1 and mSIN3A (SIN3 transcription regulator family member A), a protein involved in the regulation of histone acetylation. Competition between DNMT1 and mSIN3A, for an interaction with the TRD (transcription repression domain) of MeCP2, disrupted the MeCP2/HDAC1/mSIN3A complex in benefits of the recruitment of DNMT1 on hemi-methylated DNA and favored DNA methylation [51].

##### DNMT1/DMAP1

DMAP1 (DNMT-associated protein1) is a transcriptional co-repressor also involved in DNA methylation inheritance. DNMT1/DMAP1 interaction (via aa 12–105 of DNMT1) was involved in both early (euchromatin) and late S phase (heterochromatin) of DNA replication and in the recruitment of PCNA [3]. The DNMT1/DMAP1 complex has been shown to repress glucocorticoid receptor target genes. This silencing also required the DMAP1-mediated recruitment of the multifunctional protein DAXX (death domain-associated protein) whose roles have been previously reported in apoptosis and transcriptional repression [52]. Similarly HDAC2 could also associate with the DNMT1/DAMP1 complex in late S phase and promote gene silencing [53]. On the opposite, RGS6 (regulator of G protein signaling 6) could compete with DNMT1 for the interaction with DMAP1 and disrupt DNMT1/DMAP1 interaction [54, 55].

In spite of the absence of a clear affinity for a particular DNA sequence, each DNMT1-including complex could be associated to a different or a partially redundant DNA methylation profile. Indeed, a specific peptide-mediated disruption of DNMT1/PCNA, DNMT1/HDAC1, DNMT1/DNMT3B, or DNMT1/HP1 interactions promoted global DNA hypomethylation in astrocytes and increased tumor growth [27, 56, 57]. On the opposite, a specific inhibition of DNMT1/DMAP1 interaction increased the temolozomide response in glioma cells, suggesting that inhibition of specific DNMT-including complexes could be used in the future in combination with classical chemotherapeutic agents.

#### Maintaining DNA methylation activity and pathologies

##### DNMT1 and viral oncoproteins

As already seen above, DNA methylation deregulation is associated with tumorigenesis. An association of DNMT1 with two distinct viral oncoproteins has been reported. Both DNMT1/E1A (in adenovirus) and DNMT1/E7 (in papillomavirus) interactions increased inheritance DNA methylation. Although mechanisms governing this phenomenon are still unclear, it has been proposed that viral oncoproteins might, as already described for DNMT3L, promote DNMT1 DNA binding and SAM recruitment [58].

##### Regulation of inheritance DNA methylation activity

A deregulation of inheritance methylation activity was reported in many pathologies. Indeed, in lupus patients, a decrease of DNMT1 was mediated by the overexpression of *miRNA-21* (*microRNA*) and *miRNA-148a* that control the *DNMT1* gene expression [59]. In acute myeloid leukemia cells, *miRNA-29b* inhibited the expression of SP1 (specific protein-1, TF required for DNMT1 expression) and consequently decreased DNMT1 expression [60]. In spite of global DNA hypomethylation and a decrease in maintaining methylation activity, a decrease of DNMT1 content was rarely observed in solid tumors. Numerous post-translational modifications of DNMT1 could modulate its activity in cancers. Indeed, Casein kinase-1 induced the S146 phosphorylation of DNMT1 and decreased the DNA binding capacity of this enzyme [61]. S127 and S143 phosphorylations mediated by PKC (protein kinase C) and AKT (also called PKB, protein kinase B) were observed in glioma and provoked the disruption of DNMT1/PCNA/UHRF1 complex and a consecutive global DNA hypomethylation [27, 62]. On the opposite, S143 phosphorylation could also block the SET-7-mediated K142 methylation which normally promotes proteasomal degradation of DNMT1 [63, 64]. An increase in the stability of DNMT1 was also reported following its demethylation by LSD1 [65]. Moreover, 10 putative sites of sumoylation were reported in DNMT1 whose roles are still unclear [66].

#### Role of DNMT3A/3B in DNA methylation inheritance

In spite of their predominant role in de novo methylation, DNMT3A and DNMT3B are also involved in maintaining DNA methylation [67]. Cooperation between DNMT1 and DNMT3A, due to a partially redundant and/or a complementary maintaining activity, has been reported in post-mitotic neurons [68]. Direct interaction between N-ter regions of DNMT1 and of DNMT3A/B has been involved in this cooperation [69]. Indeed, these interactions were necessary for maintaining DNA methylation of heterochromatin in embryonic cells. Indeed, DNMT3A and/or DNMT3B invalidation(s) induced a loss of maintaining of DNA methylation in specific loci (e.g., hypomethylation of imprinted genes *IGF2* and *XIST*) and a progressive global DNA hypomethylation [70, 71]. Jeong et al. proposed a model, in which a pool of DNMT3A and DNMT3B already bound to nucleosomes in CG-rich regions could catalyzed the inheritance methylation of CpGs previously missed by DNMT1 during the reading of hemi-methylated DNA following DNA replication [72].

In cells, maintaining and de novo DNA methylation activities are not compartmentalized and evident crosstalks between these machineries have been underlined. Indeed, in colorectal cancer cells, invalidation of DNMT1 or DNMT3B had minor effects on global DNA methylation while double invalidation reduced to more than 90% the 5mC content [73, 74]. Moreover, a close cooperation between DNMT1 and DNMT3s was reported for the methylation of specific genes in cancer cells. For example, we reported that DNMT1 and DNMT3A were necessary for the methylation of the *CASP8* promoter in glioma cells [75, 76].

### Unspecific de novo DNA methylation machineries

#### De novo DNA methylation activity is mainly catalyzed by DNMT3A and DNMT3B

Mutations in the *DNMT3B* gene induce a specific hypomethylation of heterochromatin *satellite-2* sequences leading to the ICF (immunodeficiency, centromeric instability, and facial dysmorphism) syndrome. Indeed, de novo DNA methylation of CpG-rich sequences (e.g., *satellite-2* sequences) could be easily catalyzed by a processive enzyme such as DNMT3B (DNMT3B presents six additional positives charges in C-ter), whereas DNMT3A is a distributive enzyme [77]. Contrary to DNMT1 which is mainly recruited in replication loci during S phase, DNMT3A and DNMT3B are not focused to these loci. For example, during DNA replication, DNMT3B can interact with hCAP-C, E, and G (human chromosome-associated protein) and three members of the condensin complex responsible for chromosomal condensation. This suggests that DNA methylation catalyzed by DNMT3B is, at least, partially independent from DNA replication [78, 79]. Moreover, an increase of de novo DNA methylation activity of DNMT3B following its interaction with NEDDylated CUL4A (CUL4A-NEDD8) could be involved in local DNA hypermethylation and was reported in tissues (e.g., breast cancer (BC) and hepatoma) overexpressing CUL4A [80].

##### DNMT3A/DNMT3B/DNMT3L and heterochromatin

The absence of methylation of H3K4 (H3K4me0) also controls the DNMT3 recruitment on chromatin during gametogenesis and embryogenesis. Moreover, the identification of H3K4me0 by DNMT3L (via PHD domain) could also promote in a DNMT3L/DNMT3A/3B interactions manner, the recruitment of DNMT3A and DNMT3B on DNA. Crystal structure of DNMT3L/DNMT3A complexes revealed that these proteins associated in dimers or tetramers (1–2 DNMT3A linked via their C-ter region to the C-ter region of 1–2 DNMT3L) [81–85]. These complexes induced a refolding of DNMT3A that increased its DNA binding capacities and de novo methylation activity of 2- to 20-fold [77, 86–88]. The recruitment of DNMT3A/DNMT3L-including complexes was more frequent in *Alu* sequences, in the promoters of imprinted genes and in CpG-rich regions with CpG spaced from 8 to 10 pb [82, 89]. About 100 imprinted genes were described in mammals, and most of them are grouped in clusters. Although both DNMT3A and DNMT3B have been involved in the nuclear localization of DNMT3L, the DNMT3A/DNMT3L interaction seems the most important for gene imprinting, since invalidation of DNMT3A or DNMT3L alone provoked a loss of imprinting marks and gene reactivation but not the DNMT3B KO [90–92].

##### DNMT3A/B-including complexes and histones marks

As seen above for unspecific maintaining DNA methylation, de novo methylation also frequently requires cooperation between DNMTs and chromatin remodelers [93]. e.g., interaction of DNMT3A and DNMT3B with LSH (lymphoïd-specific helicase), a member of the SNF2-related family, increased the processivity of these DNMTs and their DNA binding capacities [94]. LSH invalidation, in ES cells, provoked a hypomethylation of DNA repeat elements and expression of specific genes [95, 96]. Recruitment of HDACs in the fleeting DNMT3B/LSH/DNMT1/HDCA1/HDAC2 complex could also increase the repressor activity of LSH [97]. Indeed, during oogenesis, the repression of imprinted genes was achieved by histone modifications and the co-recruitment of HDAC1 mediated by the PHD domain of DNMT3L. HDAC1 and/or HDAC2 may be also recruited by a direct interaction with DNMT3A and DNMT3B [78, 98–102].

**Fig. 3:**
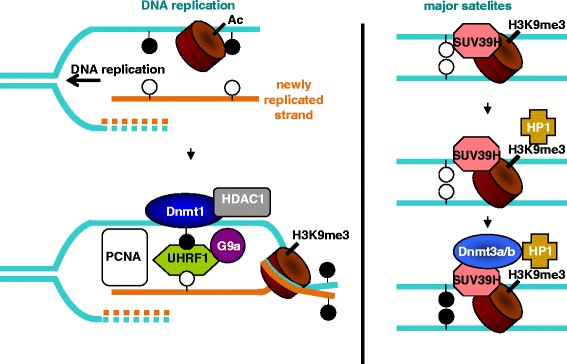
Examples of cooperation between DNA methylation machineries and proteins regulating post-translational modifications of histones. Left: Silencing of a DNA region following DNA replication. Addition of the repressive H3K9me3 mark, removal of H3 acetylation (Ac) and methylation of the new DNA strand is catalyzed by the DNMT1/UHRF1/PCNA/G9a/HDAC1 complex. Right: de novo methylation of a DNA region of heterochromatin (e.g., major satellites). The addition of the H3K9me3 repressive mark by SUV39H1 is read by HP1 and that further induces the recruitment of DNMT3A and/or DNMT3B for de novo methylation of both strands of DNA. Black circles: 5mC; white circles: C. Parental strand: blue; new synthetized strand: orange

DNMT3A could also directly read the H3K36me3 and H4R3me2 marks to complete gene repression in a DNA methylation manner (e.g., *B-GLOBIN* gene), suggesting that de novo methylation activity is also closely related to histone methylation [103, 104]. Moreover, de novo DNA methylation occurring in heterochromatin could be initiated by the SUV39H1-mediated H3K9me3 methylation which could be recognized by HP1. HP1 can finally recruit DNMT3A and/or DNMT3B for DNA methylation (Fig. 3) [42]. SUV39H1/DNMT3B interaction was mainly involved in pericentric heterochromatin methylation and not in centromeric methylation, suggesting that different mechanisms are required in regard of heterochromatin localization. On the opposite, the anchorage of DNMT3B on centromeric areas was favored by its interaction with the centromeric protein CENP-C (via the PWWP domain of DNMT3B) [105].

Similar mechanisms were also reported in euchromatin: as an example, the kinetic of *TNFα* gene silencing required (i) an initial addition of the H3K9me2/3 mark by G9a, (ii) the identification of this mark by HP1, and (iii) the silencing was then completed by the recruitment of DNMT3A and DNMT3B for DNA methylation [93, 94, 106]. Moreover, SETDB1 (SET domain bifucarted 1; also called ESET), another HMT, specific of euchromatin, could also directly interact with DNMT3A and DNMT3B, but not with DNMT1 (even if SETDB1 could be indirectly associated with DNMT1 via SETB1/MBD1 interaction), to induce gene silencing [107], e.g., the repression of the *RASSAF1A* gene required (i) addition of H3K9me3 marks on the promoter by the SETDB1/HDAC1 complex, (ii) recruitment of DNMT3A (via DNMT3A/SETBD1 and DNMT3A/HDAC1 interactions), and (iii) DNA methylation. The formation of the BRG1/G9a/DNMT3A complex was induced following stress induction in mice and responsible for the repression of the motor MYH6 gene and cardiac dysfunctions [108].

The direct methylation of the murine DNMT3A (K44me2) by G9a or by GLP (G9a-like protein) could also be recognized by MMP8 (M-phase phosphoprotein 8). The DNMT3A/MPP8/G9a(or GLP) silencing complex was predominantly recruited close to H3K9me marks [109].

##### DNMT3A/DNMT3B/UHRF1

UHRF1, the essential component of the unspecific maintaining DNA methylation activity catalyzed by the DNMT1/PCNA/UHRF1/G9a complex, is also able to interact with DNMT3A and DNMT3B independently of the presence of DNMT1 [110]. These interactions require the N-ter regions of DNMT3A and DNMT3B and the SRA domain of UHRF1 (also included in DNMT1/UHRF1 interaction). Indeed, in ES transfected cells, silencing of the exogenous CMV promoter was dependent of the presence of UHRF1, G9a, SUV39H, DNMT3A, and DNMT3B.

#### De novo DNA methylation is partially catalyzed by DNMT1

Although DNMT1 is predominantly involved in maintaining DNA methylation and that its affinity for hemi-methylated DNA is 2- to 50-fold more important than for unmethylated DNA, large high-resolution sequencing of repetitive elements or of single copy genes revealed a role of DNMT1 in de novo methylation [67]. Indeed, de novo methylation of genes frequently observed in cancers could be catalyzed by DNMT1 rather than DNMT3A or DNMT3B [111, 112]. Moreover, de novo methylation of the *D4Z4* subtelomeric repeat was dependent of DNMT1 and not DNMT3B [113]. Among the three areas of DNMT1 mapped to interact with DNA, the Zn-binding domain which recognizes methylated DNA, has been involved in de novo methylation activity of DNMT1 [114]. Recruitment of DNMT1 (via the allosteric site in the N-ter) to already methylated DNA, also increases its de novo methylation activity. Similarly, de novo DNA methylation, catalyzed by DNMT3A, promoted a consecutive increase in de novo methylation activity of DNMT1. Interaction of USP7 with DNMT1 (via USP7 C-ter domain of USP7 and DNMT1 TS domain) and UHRF1 (via USP7 TRAF-domain and UHRF1 SRA-domain) also stimulated both inheritance and de novo methylation activities of DNMT1 and stabilized UHRF1 content via its deubiquitination [115]. All of these observations strongly suggest a tight cooperation between all DNMTs in de novo methylation activity, even if putative partners involved in these processes, are largely unknown.

## Specific recruitment of DNA methylation machineries

**Fig. 4:**
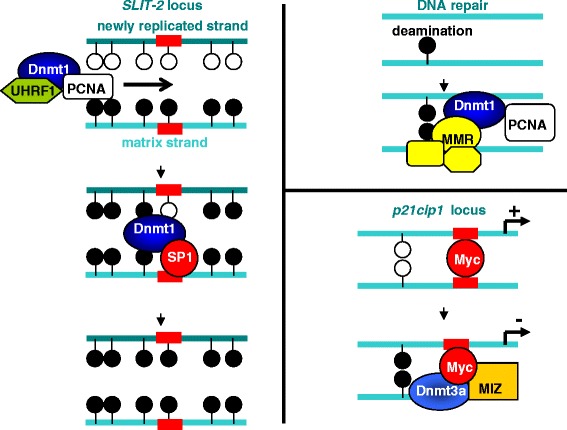
Illustration of the role different DNMT-including complexes in the maintaining DNA methylation. Left: The canonical unspecific DNMT1/UHRF1/PCNA complex processed the maintaining of DNA methylation of most of the CpGs. A cooperation with specific DNMT-including complexes, as illustrated by the SP1/DNMT1 interaction catalyzed the methylation of CpGs on particular loci (e.g., *SP1* response elements) which were not methylated by the DNMT1/UHRF1/PCNA complex [40]. Up right: DNA deamination is repaired by a cooperation between the DNA repair machinery (MMR) and DNMT1 [119]. Bottom right: in absence of MIZ, cMYC binds to its response element and activate the expression of the *p21* gene. In presence of MIZ, the ternary complex MIZ/cMYC/DNMT3A repressed the *p21* gene in a DNA methylation manner [155, 156]

As seen above, de novo methylation is crucially related to many pathologies. In cancer cells, a paradoxal global DNA hypomethylation is frequently concomitant with both local hypo and hypermethylations of genes. These defects in DNA methylation could induce TSG silencing or resistance to cells death inducers [76]. In glioma cells, DNA demethylation of promoters, following specific inhibition of DNMT1, DNMT3A, or DNMT3B, was not fully redundant suggesting the existence of different target patterns for these enzymes [116]. Indeed, the direct interaction of DNMT1 (via its TS domain) or of DNMT3A and DNMT3B (via their PWWP domain) with DNA are not very specific and only an inaccurate consensus could be measured for preferential DNMTs targeting. Favorable sequence for DNMT1-mediated methylation was associated with an absence of G in − 1 position, while DNMT3A preferentially methylated sequences with pyrimidines in − 2 and + 1 positions [117]. We predicted that DNMT1, DNMT3A, and DNMT3B respectively preferentially methylate (A/G/T)(T/G/A)(T/A/C)**CG**(T/G/A)(C/A/T)(A/T/C), (T/A/C)(A/T)(T/G/A)**CG**(T/G/C)G(G/C/A), and (A/C)(C/G/A)(A/G)**CG**T(C/G)(A/G). Others reported that CANAGCTG and CCGG(A/T)NC(C/G)C sequences were more frequently found in methylated genes, following overexpression of DNMT3A and DNMT3, respectively [118]. However, recurrent profiles of de novo methylation were observed in cancers, suggesting that DNMTs could be specifically targeted on particular loci. The idea that “targetors” able to target DNA methylation machineries on appropriate sites has emerged since 10 years. The precise mechanisms explaining how specific de novo methylation occurs are still poorly understood. However, the discovery of particular DNMT-including complexes able to be recruited on specific loci and the fact that a single CG methylation among 300 pb in a promoter was enough to drop gene expression argues that specific de novo methylation could be mainly regulated via DNMT-“targetors” interactions (Fig. 4).

### DNA repair requires specific DNA methylation

The mechanisms involved in DNA repair, following DNA breakages, abasic sites formation or inappropriate matching, have been intensively investigated. However, the question of how epigenetic marks are repaired is still unclear. Indeed, 5mC are mutational hotspots, as the non-correction of a 5mC deamination lead to the incorporation of T:G mismatches in DNA. Even if 5mC deamination is properly corrected by a C, loss of methylation can potentially upregulate the expression of the concerned genes. The association of DNMTs with DNA repair machineries may also occasionally provoke de novo methylation and aberrant gene silencing. Indeed, DNMT1 could interact with the MMR (mismatch repair), the major DNA repair complex. DNMT1 recruitment on DNA break was independent of DNA replication and S phase, but was still mediated by PCNA [119]. Moreover, the recruitment of PCNA on DSB (double-strand break) DNA also required both PCNA/DMAP1 and PCNA/MUTS interactions (MUTS is a member of MMR and presents a strong affinity for hemi-methylated DNA) [120]. Cell irradiation also provoked an accumulation of DNMT1 but not of DNMT3A or DNMT3B.

DNMT3A or DNMT3B was also involved in DNA methylation following DNA repair, via a direct association with MBD4 (methyl-CpG-binding domain protein 4) and TDG (G/T mismatch-specific thymidine DNA glycosylase), two enzymes involved in the (base excision repair) BER complex [121]. SIRT1 can also favor the recruitment of DNMT3B and members of the polycomb group (PcG) complex on DSB DNA [122–124]. These interactions involved both the PWWP and catalytic domains of DNMT3A and DNMT3B and increased TDG activity while TDG concomitantly regulated DNMT3 activity. Once the DNA base is repaired, BER proteins detached from DNA and DNMT3s might methylate the new incorporated cytosine. Finally, DNMT1 and the TDG/DNMT3A/DNMT3B complex could also cooperate during active demethylation, independent of the presence of DNA damage. For example, a dynamic methylation/demethylation process, involving this complex, was reported on the *PS2* promoter [125].

### Specific DNMT recruitment mediated by transcriptional activators or repressors

In many cancers, the activation of the oncogene *Ras* induces a variety of events in favor of tumorigenesis, and among them, the specific silencing of a particular panel of genes. For example, the repression of the proapoptotic gene *Fas* was specifically mediated by a coordination of different complexes including 28 RESEs (Ras epigenetic effectors) leading to the recruitment of DNMT1 and the methylation of *FAS* promoter [126].

#### Recruitment of DNA methylation machineries by polycomb proteins

The polycomb group (PcG) system is composed of four interdependent multi-protein repressor complexes (PRC1 and 2/3/4) involved in the regulation of homeotic genes during development and chromatin remodeling in stem cells. Since some particular areas of DNA are controlled by PcG and are generally highly methylated, it has been suggested that PcG complexes are connected to DNA methylation machineries, to silence specific loci. PRC1–4 multi-protein complexes sequentially inhibit *HOX* genes expression by (i) inducing PRC2-mediated ubiquitinylation of H2AK119, (ii) inducing the PRC1-mediated H3K27me3 mark, (iii) a direct interaction of BMI1 (which catalyzes the ubiquitin ligase activity of PRC1) with DMAP1 leading to the recruitment of DNMT1 and the silencing of particular genes [127, 128]. A similar complex composed of DNMT1, NSPc1 (nervous system polycomb), a homolog of BMI1, and EZH2 (enhancer of zest homolog 2; H3K27 methyltransferase, member of PRC2) also specifically silenced *HOX* genes [129, 130]. DNA hypermethylation observed in colon cancer could be partially regulated by PcG/DNMTs interactions. Indeed, 47% of genes regulated by DNMT3B in these tumors also bound PRC1 or PRC2 [131]. In ES cells, the specific de novo methylation of the *MYT1* promoter was dependent of interactions between DNMT3A or DNMT3B (via their PHD domain) and PRC components, and the recruitment of the complex on the *MYT* promoter was performed in an EZH2-dependent manner. Interestingly, in these cells, the EZH2-dependent recruitment of DNMT3A was associated with H3K27me3 but not always with DNA methylation suggesting new roles for DNMT3A on gene repression independently of de novo methylation [132].

The presence of transcriptional repressors in DNMT-including complexes may explain some specific targeting of DNMTs. Indeed, the complete silencing of the *OCT-4* gene which occurs during development required (i) the local G9a-mediated H3K9 methylation and (ii) the recruitment of MBD2, MBD3, and GCNF/DNMT3A/DNMT3B complex on specific RAREs boxes specifically recognized by the repressor GCNF (germ cell nuclear factor) [133, 134]. Moreover, the helicase WRNp (Werner protein) which accumulated in *OCT-4* promoter and interacted with G9a also favored a direct interaction of G9a with DNMT3A and DNMT3B (via ANK domain of G9a) [135].

Interaction of DNMT3A with the transcriptional repressor RP58 also promoted the recruitment of DNMT3A on *RP58* response elements although local DNA methylation was not observed [101]. Additional repressor/DNMTs interactions could also explain the specific gene silencing frequently observed in numerous pathologies: Indeed, the HBX (hepatitis B virus x protein) mediated specific repression of genes (e.g., *IL-4* or *IGFBP-3*) and required direct interactions of HBX with both DNMT3A and HDAC1 [136, 137].

#### rDNA methylation

The specific silencing of gene coding for rRNA (rDNA) requires both DNA methylation and chromatin remodeling and is orchestrated by a putative complex including TIP5 (TTF-I interacting protein 5), SNF2h, HDCA1, DNMT1, and DNMT3B. Interaction of TIP-5 with H4K16ac marks on rDNA was required for the recruitment of HDAC1, DNMT1, and SNF2h and for the consecutive local deacetylation and DNA methylation in these promoters [138, 139].

### When transcriptional factors are required for epigenetic silencing

#### Specific recruitment of DNMT1 by TFs

Roles of TFs in DNA methylation are still unclear. However, in regard to recent data of literature, it appeared that DNMTs are able to “use” a TF as a co-repressor.

DNMT1 activity is processive, but we reported that methylation of several CpGs within the same promoter could be catalyzed by different DNMT1-including complexes. Indeed, maintaining of DNA methylation of some CpGs in the *SLIT2* promoter was mainly processed by the canonical DNMT1/PCNA/UHRF1 complex, but methylation of specific CpGs localized in or near a SP1 box, within the promoter, was methylated by the DNMT1/SP1 complex (Fig. 4) [140]. The major DNMT1/PCNA/UHRF1 complex is mainly formed and recruited during the S phase of cell cycle, but additional DNMT1/TFs interactions are associated with different phases. For example, DNMT1 could interact predominantly with SP1 during G1 and G2 phases, while DNMT1/P53 and DNMT1/E2F3 interactions were mainly observed during G2 and S/G2 phases respectively in U251 cells [141].

##### DNMT1/P53

Activation of the TF P53 increased the expression of a large group of genes while surprisingly, a fraction of genes with a P53 box where repressed [142]. A ternary repressor DNMT1/P53/HDCA1 complex was able to repress, in a DNA methylation manner, the expression of specific genes (e.g., *SURVIVIN*) by catalyzing both DNA methylation and histone deacetylation, on specific DNA loci recognized by P53. Repressive capacities of DNMT1/P53-including complexes are also controlled by additional regulators. Indeed, following DNA damage, DNMT1/P53-mediated silencing of the *SURVIVIN* gene was dependent of NBS1 (Nijmegen breakage syndrome)/DNMT1 interaction [143]. Likewise, the P53-dependent repression of *CDC25C* required SP1 (specificity protein-1)/P53 interaction, allowing the recruitment of DNMT1 close to *SP1* and *P53* response elements [144]. P53/mSIN3a and P53/HDACs interactions were also involved in P53-mediated gene silencing. Interestingly, the indirect interaction of the mutated P53 with DNMT1/HDAC1/HDAC2/MeCP2 complex was also implicated in *ERα* (estrogen receptor: *ESR1*) silencing in MDA-MB-468BC cells, suggesting that abnormal TFs may mediate specific DNA methylation in cancers [145].

##### DNMT1/RUNX1-MTG8

Modified TFs (punctual mutations, chimerical proteins, and specific post-translational modifications) or an increase expression of a particular TF may contribute to generate new TF/DNMTs interactions or to favor pre-existing interactions in pathologic tissues and therefore to induce specific DNA methylation. Indeed, the t(8;21)(q22.q22) translocation, which was frequently observed in acute myeloid leukemia, induces the formation of the chimerical protein RUNX1 (runt-related transcription factor 1, also called AML1 or CBFA2) -MTG8 (ETO, CBFA2T1) which mimics an oncogenic TF. In normal cells, RUNX1 bound to the enhancer sequence TGT/CGGT whereas MTG8 is a transcriptional repressor able to interact with other co-repressors. Direct or indirect DNMT1/RUNX1-MTG8 interaction was observed in a complex including the co-repressors HDACs, mSIN3a and N-Cor and all components synergistically silenced some specific genes (e.g., *IL-3*) [146, 147].

##### STAT3/DNMT and cancer

The repression of the *PTPN6* gene was mediated by the recruitment of DNMT1/STAT3(phosphorylated)/HDAC1 complex on STAT3 boxes n *PTPN6* promoter [148]. Moreover, STAT3 acetylation (K685ac), which increased in melanoma, triple negative BC, or in colon cancer compared to normal tissue, was also associated to a specific profile of DNA methylation. The mutated STAT3 K685R inhibited DNMT1/STAT3 interaction and restored the expression of these genes. Indeed, specific inhibition of DNMT1/STAT3 interaction using peptides competitors also significantly decreased glioma-cell proliferation [49].

##### HESX/DNMT1

During development, many genes are thinly and kinetically regulated. One of the TF, controlling this timing is HESX1 (HESX homeobox 1), which mediates the repression of HESX1-target genes by both recruiting co-repressors (such as TLE1 or N-Cor) and by specific DNA methylation in a HESX1/DNMT1 interaction manner.

##### Indirect interaction with TF

The target of DNMT1-including complexes on specific TF-response elements can also be mediated by indirect interactions with TFs. Indeed, the presence of the transcriptional repressor DAXX lead to the silencing of ReIB target genes which are normally activated by this TF. This DNA methylation repression is mainly mediated by the indirect recruitment of DNMT1 via the DAXX/DNMT1 and DAXX/RelB interactions and is completed by the further recruitment of HDAC2 on these promoters [149]. Similarly, P53 was also required for the recruitment of DAXX and DNMT1 on *RASSAF1A* promoter and its methylation in lymphoblastic leukemia [150].

#### Specific recruitment of DNMT3 and DNMT3B by TFs

TFs are also capable of inducing the specific recruitment of DNMT3A and DNMT3B in DNA. DNMT3A can interact with P53 (via its C-ter) leading to the repression of P53-regulated genes (e.g., *p21*) although the existence of P53/DNMT3A-mediated de novo methylation was not clearly demonstrated [151]. On the opposite, in the lymphocytes lineage, the recruitment on purine-rich sequences of the DNMT3A/DNMT3B/PU.1 complex (which requires the interaction of the ETS domain of PU.1 with the ATRX domain of DNMT3s) induced the DNA methylation of PU.1-regulated genes such as *p16(INK4a*). Silencing of the target genes was then completed by histone modifications mediated by the PU.1/mSIN3a/HDAC/MeCp2 complex [152]. During transformation of HS cells following overexpression of the oncoprotein EVI1, DNMT3A and DNMT3B could interact with EVI1 and therefore specifically methylate the *miRNA-124-3* promoter [153]. Similarly, interaction of DNMT3A with ISGF3 or AP2a, and DNMT3B with CREB1, ELK1 or PPARg may be important for the repression of genes normally controlled by these TFs. For example, inhibition of the DNMT3A/ISGF3ϒ interaction increased the response to temozolomide in glioma models [154]. A direct interaction of c-MYC with DNMT3A and DNMT3B was responsible for the recruitment of DNMT3 on a *c-MYC* box in *CCND1* promoter, in a MIZ1-dependent manner. On the opposite, in the absence of MYZ1, c-MYC recruitment on *c-MYC* response element induced the transcriptional activation of *CCND1* [155, 156]. Similarly, the ZEB-1 (an epithelial to mesenchymal transition-TF)/HDAC1/DNMT3A complex has been involved in the specific repression of Neurogenin 3 gene [157]. The same complex was also involved in the ESR1 gene repression following its recruitment on E2-boxes identified by ZEB-1 [158]. In leukemia, the frequent translocation of t(15;17) leads to the formation of the PML-RARα fusion protein. Recruitment of PML-RARα on *RARβ2* promoter induced the recruitment of DNMT1 and DNMT3A by direct interactions [159].

#### E2F family and DNMTs recruitment

The E2F family members regulate the expression of many genes during cell cycle by interacting to *E2F* response elements. Interaction of E2F6 with DNMT3B but not with DNMT3A was required for specific homeotic silencing [156, 160]. The recruitment of the DNMT1/HDAC1/RB/E2F1 complex was dependent of the presence of *E2F* response elements, and this complex controlled the expression of genes normally activated during middle G1 to late S phase [161]. The DNMT1/p130-RB2/E2F4/E2F5/HDAC1/SUV39H complex was also involved in silencing of the *ER-a* gene [45, 46]. Moreover, the LANA antigen (Kaposi’s sarcoma-associated herpes virus LANA), which indirectly activates some genes in an E2F activation manner, could also specifically silence other genes (e.g., *13H CADHERIN)* by inducing LANA/DNMT3A/E2F interaction-mediated de novo methylation [162].

#### Redundancy or specificity of TF/DNMTs interactions

**Fig. 5:**
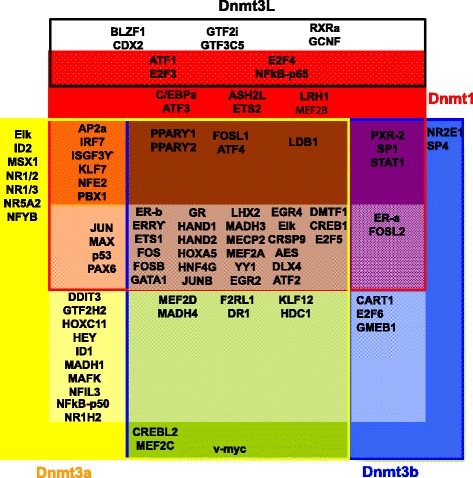
Putative TF/DNMT interactions. In vitro experiments with transcriptional factors arrays and recombinant DNMT1, DNMT3A, DNMT3B or DNMT3L revealed that some TFs can potentially interact with several or only one DNMT. White: TFs interacting only with DNMT3L; red: TFs interacting only with DNMT1; yellow: TFs interacting only with DNMT3A; blue: TFs interacting only with DNMT3B; green (blue+yellow): TFs interacting with both DNMT3A and DNMT3B; orange (red + yellow): TFs interacting with both DNMT3A and DNMT1; purple (red + blue): TFs interacting with both DNMT3B and DNMT1; light red: TFs interacting with both DNMT1 and DNMT3L; brown (red + blue + yellow): TFs interacting with DNMT1, DNMT3A and DNMT3B; light yellow (yellow + white): TFs interacting with both DNMT3A and DNMT3L; light blue (blue + white): TFs interacting with both DNMT3B and DNMT3L; light green (green + white): TFs interacting with DNMT3A, DNMT3B and DNMT3L; light orange (orange + white): TFs interacting with DNMT1, DNMT3A and DNMT3L; light purple (purple + white): TFs interacting with DNMT1, DNMT3BDNMT3L; light brown (brown + white): TFs interacting with all DNMTs. Adapted from [140, 156, 163]

Little is currently known about the specificity of each DNMT for one or another protein partner. Although some redundancy may explain that invalidation of one DNMT could sometimes be balanced by another one (P53 can interact with both DNMT1 and DNMT3A), the analysis of cell-validated or in vitro putative TF/DNMT interactions revealed that some interactions are specific to one DNMT [140, 156]. As illustrated in Fig. 5, 29 TFs or co-repressors tested, potentially interact with the 3 DNMTs (e.g., the MBD protein MeCP2 or the TFs GATA1, HAND1, and HAND2), while others are restricted to one or two of these enzymes (9 only with DNMT1, 17 only with DNMT3A, 5 only with DNMT3B, 10 with both DNMT1 and 3a, 5 with both DNMT1 and 3b, 9 with both DNMT3A and 3b). We could not predict interaction with any DNMT for 15 additional TFs tested. One interesting example is the E2F family. E2F5 can potentially interact with all three DNMTs while E2F4 and E2F6 can only interact with DNMT1 and DNMT3B, respectively. Specific pattern of expression of some TFs may limit their role on DNA methylation to specific tissues. For example, the putative DNA methylation activity of the DNMT3A/NR5A2 complex is probably confined to pancreatic cells, since NR5A2 is more abundant in pancreas. DNMT3L which is catalytically inactive is also capable of interacting with TFs which are similar or different from TFs interacting with DNMT3A and/or DNMT3B (Fig. 5) [163]. Moreover, the recruitment of a DNMT3L/TF-including complex on a specific locus may also regulate the specific methylation of this sequence by mediating the further recruitment of DNMT3A or DNMT3B. This has been illustrated by the specific methylation of the promoter *TRAF1* by the indirect recruitment of DNMT3A and DNMT3B via the complex DNMT3L/NFKBp65.

## Conclusion

**Fig. 6:**
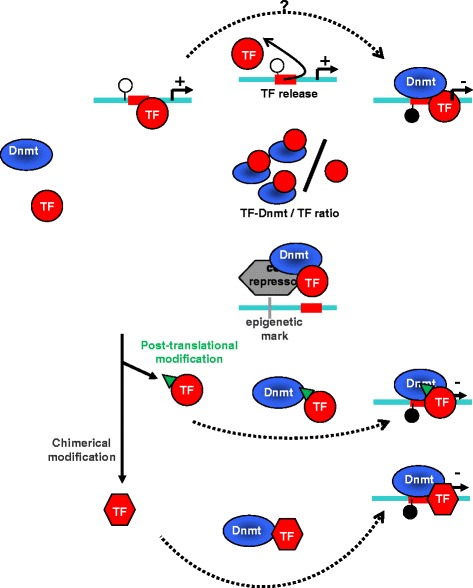
TF: a balance between activator and repressor. The switch between the activator role or the repressor role of a TF, as that is found in DNMT-including complexes to specificallly methylate some DNA sequences could be explained by different hypothesis: (i) a modulation of the TF/DNMT versus free TF ratio [157, 161], (ii) the stabilization of the TF/DNMT interaction by another co-repressor (e.g., HMTs, HDACs) [149–152], and (iii) post-translation modifications of TF in favor of DNMT/TF interaction; a genetic (fusion protein) or 3D structure modification of the TF in favor of DNMT/TF interaction [49, 145–147]

Epigenetic silencing appears thinly regulated and orchestrated and frequently requires the presence of several DNMT-including complexes, polycomb proteins and HDACs, interacting together in a specific kinetic. A list of the main repressors directly or indirectly interacting with DNMTs is proposed in Table 1. For example, epigenetic silencing of the *NY-ESO1* gene in glioma and mesothelioma cells required the sequential recruitment of three independent complexes: (i) HDAC1/mSIN3a/NCOR complex which deacetylates the promoter, (ii) DNMT3B/HDAC1/EGR1 complex which induces a local DNA methylation and increases histone deacetylation, and (iii) DNMT1/PCNA/UHRF1/G9a complex which maintains DNA methylation and introduces the H3K9me2 repressive mark [164]. The question related to the switch between transcriptional to “repression activity” of a TF is still unclear. Contrary to TF-mediated maintaining of DNA methylation in promoters, already repressed, the role of TF in specific TF-mediated de novo methylation is crucial for the control of gene expression. Ratio between free TF versus TF-DNMT complex or association with additional co-repressor in multi-protein complexes could determine the nature of the activity (activator or “repressor”) of each TF able to associate with a DNMT. For example, it has been proposed that, following DNA damages, an increase of P53 content could incline towards the P53/DNMT1 interaction and DNA methylation. Indeed, PU.1/DNMT3A/DNMT3B interactions may be favored by high amounts of PU.1 in leukemia [144]. A second hypothesis could incriminate the accessibility of the TF-response element: Detachment of a free TF from its response element may allow the specific recruitment of the DNMT/TF complex. Finally, post-translational modifications of TFs could also regulate the binding capacities and stability of TFs/DNMT-including complexes and may involve enzymes required for histones modifications (STATs, HDACs) (Fig. 6). The understanding of these mechanisms will constitute a great challenge to determine the kinetic of the events, the possible correlations between TF expressions and specific gene repressions, and/or the existence of specific DNA sequences that could be more sensible to TF-mediated methylation.

**Table 1:**
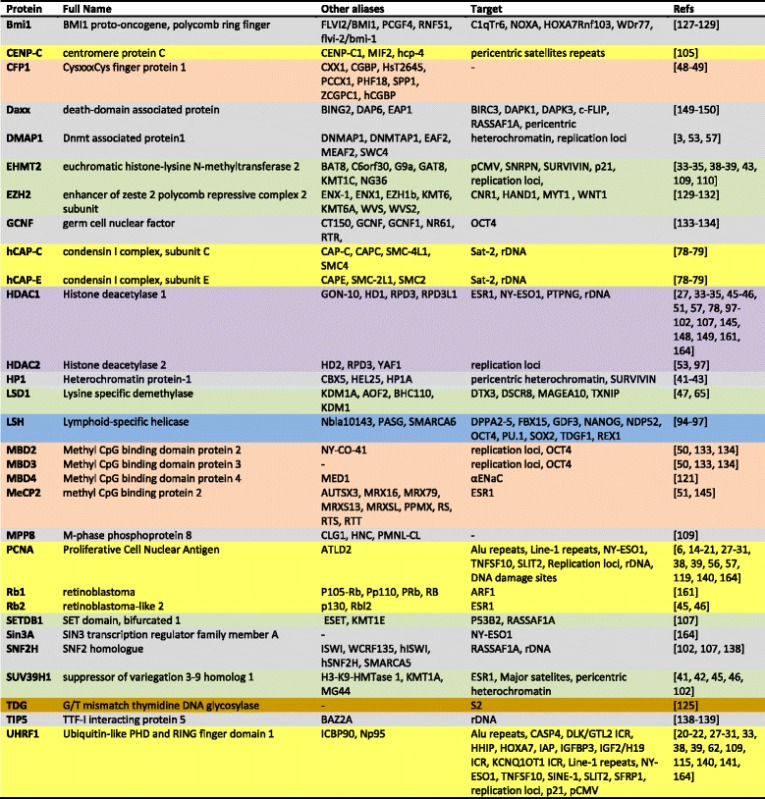
List of the main co-repressors directly or indirectly interacting with DNMTs

Aliases (source NCBI), full names, and targets are summarized. Protein functions are calssified with different colors: gray: scaffold and connector proteins; yellow: proteins involved in DNA replication and cell division; salmon: proteins interacting with methylated DNA; green: histone methylases or histones demethylases; purple: histone deacetylase; brown: DNA repair proteins

## Abbreviations

aa: Amino acids
BAH domains: Adjacent homology domain
BC: Breast cancer
BER: Base excision repair
CFP1: CysxxCys finger protein 1
DNMT: DNA methyl transferase
ERα: Estrogen receptor
EZH2: Enhancer of zest homolog 2
GCNF: Germ cell nuclear factor
HBX: Hepatitis B virus x protein
hCAP: Human chromosome associated protein
HESX1: HESX homeobox 1
HMT: Histone methyl transferase
HP1: Heterochromatin protein-1
ICF: Immunodeficiency, centromeric instability, and facial dysmorphism
LSD1: Lysine-specific demethylase 1A
LSH: Lymphoïd-specific helicase
MBD: Methyl CpG-binding domain protein
*miRNA*: MicroRNA
mSIN3A: SIN3 transcription regulator family member A
NBS1: Nijmegen breakage syndrome
NSPc1: Nervous system polycomb
PcG: Polycomb group
PCNA: Proliferative cell nuclear antigen
PKC: Protein kinase C
RESEs: Ras epigenetic effectors
RFTS: Replication focus targeting sequence
RUNX1: Runt-related transcription factor 1
SETDB1: SET domain bifucarted 1
SP1: Specific protein
SRA: SET and RING
TRD: Transcription repression domain
UHRF1: Ubiquitin-like PHD and RING finger domain 1
WRNp: Werner protein

## References

[1] Goll MG, Kirpekar F, Maggert KA, Yoder JA, Hsieh CL (2006). Methylation of tRNAAsp by the DNA methyltransferase homolog DNMT2. Science.

[2] Okano M, Xie S, Li E (1998). DNMT2 is not required for de novo and maintenance methylation of viral DNA in embryonic stem cells. Nucleic Acids Res.

[3] Negishi M, Chiba T, Saraya A, Miyagi S, Iwama A (2009). Dmap1 plays an essential role in the maintenance of genome integrity through the DNA repair process. Genes Cells.

[4] Margot JB, Ehrenhofer-Murray AE, Leonhardt H (2003). Interactions within the mammalian DNA methyltransferase family. BMC Mol Biol.

[5] Goyal R, Rathert P, Laser H, Gowher H, Jeltsch A (2007). Phosphorylation of serine-515 activates the mammalian maintenance methyltransferase Dnmt1. Epigenetics.

[6] Vilkaitis G, Suetake I, Klimasauskas S, Tajima S (2005). Processive methylation of hemimethylated CpG sites by mouse Dnmt1 DNA methyltransferase. J Biol Chem.

[7] Bacolla A, Pradhan S, Roberts RJ, Wells RD (1999). Recombinant human DNA (cytosine-5) methyltransferase. II. Steady-state kinetics reveal allosteric activation by methylated DNA. J Biol Chem.

[8] Okano M, Bell DW, Haber DA, Li E (1999). DNA methyltransferases Dnmt3a and Dnmt3b are essential for de novo methylation and mammalian development. Cell.

[9] Pradhan S, Bacolla A, Wells RD, Roberts RJ (1999). Recombinant human DNA (cytosine-5) methyltransferase. I. Expression, purification, and comparison of de novo and maintenance methylation. J Biol Chem.

[10] Hermann A, Goyal R, Jeltsch A (2004). The Dnmt1 DNA-(cytosine-C5)-methyltransferase methylates DNA processively with high preference for hemimethylated target sites. J Biol Chem.

[11] Pradhan M, Esteve PO, Chin HG, Samaranayke M, Kim GD (2008). CXXC domain of human DNMT1 is essential for enzymatic activity. Biochemistry.

[12] Fellinger K, Rothbauer U, Felle M, Langst G, Leonhardt H (2009). Dimerization of DNA methyltransferase 1 is mediated by its regulatory domain. J Cell Biochem.

[13] Vertino PM, Sekowski JA, Coll JM, Applegren N, Han S (2002). DNMT1 is a component of a multiprotein DNA replication complex. Cell Cycle.

[14] Shimamura S, Ishikawa F (2008). Interaction between DNMT1 and DNA replication reactions in the SV40 in vitro replication system. Cancer Sci.

[15] Bestor TH (1992). Activation of mammalian DNA methyltransferase by cleavage of a Zn binding regulatory domain. EMBO J.

[16] Spada F, Haemmer A, Kuch D, Rothbauer U, Schermelleh L (2007). DNMT1 but not its interaction with the replication machinery is required for maintenance of DNA methylation in human cells. J Cell Biol.

[17] Iida T, Suetake I, Tajima S, Morioka H, Ohta S (2002). PCNA clamp facilitates action of DNA cytosine methyltransferase 1 on hemimethylated DNA. Genes Cells.

[18] Chuang LS, Ian HI, Koh TW, Ng HH, Xu G (1997). Human DNA-(cytosine-5) methyltransferase-PCNA complex as a target for p21WAF1. Science.

[19] Schermelleh L, Haemmer A, Spada F, Rosing N, Meilinger D (2007). Dynamics of Dnmt1 interaction with the replication machinery and its role in postreplicative maintenance of DNA methylation. Nucleic Acids Res.

[20] Bostick M, Kim JK, Esteve PO, Clark A, Pradhan S (2007). UHRF1 plays a role in maintaining DNA methylation in mammalian cells. Science.

[21] Sharif J, Muto M, Takebayashi S, Suetake I, Iwamatsu A (2007). The SRA protein Np95 mediates epigenetic inheritance by recruiting Dnmt1 to methylated DNA. Nature.

[22] Achour M, Jacq X, Ronde P, Alhosin M, Charlot C (2008). The interaction of the SRA domain of ICBP90 with a novel domain of DNMT1 is involved in the regulation of VEGF gene expression. Oncogene.

[23] Arita K, Ariyoshi M, Tochio H, Nakamura Y, Shirakawa M (2008). Recognition of hemi-methylated DNA by the SRA protein UHRF1 by a base-flipping mechanism. Nature.

[24] Avvakumov GV, Walker JR, Xue S, Li Y, Duan S (2008). Structural basis for recognition of hemi-methylated DNA by the SRA domain of human UHRF1. Nature.

[25] Hashimoto H, Horton JR, Zhang X, Bostick M, Jacobsen SE (2008). The SRA domain of UHRF1 flips 5-methylcytosine out of the DNA helix. Nature.

[26] Qian C, Li S, Jakoncic J, Zeng L, Walsh MJ (2008). Structure and hemimethylated CpG binding of the SRA domain from human UHRF1. J Biol Chem.

[27] Hervouet E, Lalier L, Debien E, Cheray M, Geairon A (2010). Disruption of Dnmt1/PCNA/UHRF1 interactions promotes tumorigenesis from human and mice glial cells. PLoS One.

[28] Hervouet E LL, Debien E, Cheray M, Geairon A, Rogniaux H, Valette FM, Cartron PF Tumour induction by disruption of the Dnmt1, PCNA, UHRF1 interactions. Nature Precedings 2008 hdl:10101/npre.12008.12509.10101.

[29] Robert MF, Morin S, Beaulieu N, Gauthier F, Chute IC (2003). DNMT1 is required to maintain CpG methylation and aberrant gene silencing in human cancer cells. Nat Genet.

[30] Gaudet F, Hodgson JG, Eden A, Jackson-Grusby L, Dausman J (2003). Induction of tumors in mice by genomic hypomethylation. Science.

[31] Jaenisch R, Bird A (2003). Epigenetic regulation of gene expression: how the genome integrates intrinsic and environmental signals. Nat Genet.

[32] Chen T, Hevi S, Gay F, Tsujimoto N, He T (2007). Complete inactivation of DNMT1 leads to mitotic catastrophe in human cancer cells. Nat Genet.

[33] Fuks F, Burgers WA, Brehm A, Hughes-Davies L, Kouzarides T (2000). DNA methyltransferase Dnmt1 associates with histone deacetylase activity. Nat Genet.

[34] Xin Z, Tachibana M, Guggiari M, Heard E, Shinkai Y (2003). Role of histone methyltransferase G9a in CpG methylation of the Prader-Willi syndrome imprinting center. J Biol Chem.

[35] Sharma S, Gerke DS, Han HF, Jeong S, Stallcup MR (2012). Lysine methyltransferase G9a is not required for DNMT3A/3B anchoring to methylated nucleosomes and maintenance of DNA methylation in somatic cells. Epigenetics Chromatin.

[36] Rottach A, Frauer C, Pichler G, Bonapace IM, Spada F (2010). The multi-domain protein Np95 connects DNA methylation and histone modification. Nucleic Acids Res.

[37] Liu X, Gao Q, Li P, Zhao Q, Zhang J (2013). UHRF1 targets DNMT1 for DNA methylation through cooperative binding of hemi-methylated DNA and methylated H3K9. Nat Commun.

[38] Esteve PO, Chin HG, Smallwood A, Feehery GR, Gangisetty O (2006). Direct interaction between DNMT1 and G9a coordinates DNA and histone methylation during replication. Genes Dev.

[39] Kim JK, Esteve PO, Jacobsen SE, Pradhan S (2009). UHRF1 binds G9a and participates in p21 transcriptional regulation in mammalian cells. Nucleic Acids Res.

[40] Robertson AK, Geiman TM, Sankpal UT, Hager GL, Robertson KD (2004). Effects of chromatin structure on the enzymatic and DNA binding functions of DNA methyltransferases DNMT1 and Dnmt3a in vitro. Biochem Biophys Res Commun.

[41] Lehnertz B, Ueda Y, Derijck AA, Braunschweig U, Perez-Burgos L (2003). Suv39h-mediated histone H3 lysine 9 methylation directs DNA methylation to major satellite repeats at pericentric heterochromatin. Curr Biol.

[42] Fuks F, Hurd PJ, Deplus R, Kouzarides T (2003). The DNA methyltransferases associate with HP1 and the SUV39H1 histone methyltransferase. Nucleic Acids Res.

[43] Smallwood A, Esteve PO, Pradhan S, Carey M (2007). Functional cooperation between HP1 and DNMT1 mediates gene silencing. Genes Dev.

[44] Espada J, Ballestar E, Fraga MF, Villar-Garea A, Juarranz A (2004). Human DNA methyltransferase 1 is required for maintenance of the histone H3 modification pattern. J Biol Chem.

[45] Macaluso M, Cinti C, Russo G, Russo A, Giordano A (2003). pRb2/p130-E2F4/5-HDAC1-SUV39H1-p300 and pRb2/p130-E2F4/5-HDAC1-SUV39H1-DNMT1 multimolecular complexes mediate the transcription of estrogen receptor-alpha in breast cancer. Oncogene.

[46] Rai K, Nadauld LD, Chidester S, Manos EJ, James SR (2006). Zebra fish Dnmt1 and Suv39h1 regulate organ-specific terminal differentiation during development. Mol Cell Biol.

[47] Clements EG, Mohammad HP, Leadem BR, Easwaran H, Cai Y (2012). DNMT1 modulates gene expression without its catalytic activity partially through its interactions with histone-modifying enzymes. Nucleic Acids Res.

[48] Butler JS, Lee JH, Skalnik DG (2008). CFP1 interacts with DNMT1 independently of association with the Setd1 Histone H3K4 methyltransferase complexes. DNA Cell Biol.

[49] Cheray M, Nadaradjane A, Bonnet P, Routier S, Vallette FM (2014). Specific inhibition of DNMT1/CFP1 reduces cancer phenotypes and enhances chemotherapy effectiveness. Epigenomics.

[50] Tatematsu KI, Yamazaki T, Ishikawa F (2000). MBD2-MBD3 complex binds to hemi-methylated DNA and forms a complex containing DNMT1 at the replication foci in late S phase. Genes Cells.

[51] Kimura H, Shiota K (2003). Methyl-CpG-binding protein, MeCP2, is a target molecule for maintenance DNA methyltransferase, Dnmt1. J Biol Chem.

[52] Muromoto R, Sugiyama K, Takachi A, Imoto S, Sato N (2004). Physical and functional interactions between Daxx and DNA methyltransferase 1-associated protein, DMAP1. J Immunol.

[53] Rountree MR, Bachman KE, Baylin SB (2000). DNMT1 binds HDAC2 and a new co-repressor, DMAP1, to form a complex at replication foci. Nat Genet.

[54] Xin H, Yoon HG, Singh PB, Wong J, Qin J (2004). Components of a pathway maintaining histone modification and heterochromatin protein 1 binding at the pericentric heterochromatin in mammalian cells. J Biol Chem.

[55] Liu Z, Fisher RA (2004). RGS6 interacts with DMAP1 and DNMT1 and inhibits DMAP1 transcriptional repressor activity. J Biol Chem.

[56] Hervouet E, Hulin P, Vallette FM, Cartron PF (2011). Proximity ligation in situ assay for monitoring the global DNA methylation in cells. BMC Biotechnol.

[57] Cheray M, Pacaud R, Nadaradjane A, Vallette FM, Cartron PF (2013). Specific inhibition of one DNMT1-including complex influences tumor initiation and progression. Clin Epigenetics.

[58] Burgers WA, Blanchon L, Pradhan S, de Launoit Y, Kouzarides T (2007). Viral oncoproteins target the DNA methyltransferases. Oncogene.

[59] Pan W, Zhu S, Yuan M, Cui H, Wang L (2010). MicroRNA-21 and microRNA-148a contribute to DNA hypomethylation in lupus CD4+ T cells by directly and indirectly targeting DNA methyltransferase 1. J Immunol.

[60] Garzon R, Liu S, Fabbri M, Liu Z, Heaphy CE (2009). MicroRNA-29b induces global DNA hypomethylation and tumor suppressor gene reexpression in acute myeloid leukemia by targeting directly DNMT3A and 3B and indirectly DNMT1. Blood.

[61] Sugiyama Y, Hatano N, Sueyoshi N, Suetake I, Tajima S (2010). The DNA-binding activity of mouse DNA methyltransferase 1 is regulated by phosphorylation with casein kinase 1delta/epsilon. Biochem J.

[62] Lavoie G, Esteve PO, Laulan NB, Pradhan S, St-Pierre Y (2011). PKC isoforms interact with and phosphorylate DNMT1. BMC Biol.

[63] Esteve PO, Chang Y, Samaranayake M, Upadhyay AK, Horton JR (2011). A methylation and phosphorylation switch between an adjacent lysine and serine determines human DNMT1 stability. Nat Struct Mol Biol.

[64] Esteve PO, Chin HG, Benner J, Feehery GR, Samaranayake M (2009). Regulation of DNMT1 stability through SET7-mediated lysine methylation in mammalian cells. Proc Natl Acad Sci U S A.

[65] Wang J, Hevi S, Kurash JK, Lei H, Gay F (2009). The lysine demethylase LSD1 (KDM1) is required for maintenance of global DNA methylation. Nat Genet.

[66] Lee B, Muller MT (2009). SUMOylation enhances DNA methyltransferase 1 activity. Biochem J.

[67] Arand J, Spieler D, Karius T, Branco MR, Meilinger D (2012). In vivo control of CpG and non-CpG DNA methylation by DNA methyltransferases. PLoS Genet.

[68] Feng J, Zhou Y, Campbell SL, Le T, Li E (2010). Dnmt1 and Dnmt3a maintain DNA methylation and regulate synaptic function in adult forebrain neurons. Nat Neurosci.

[69] Kim GD, Ni J, Kelesoglu N, Roberts RJ, Pradhan S (2002). Co-operation and communication between the human maintenance and de novo DNA (cytosine-5) methyltransferases. EMBO J.

[70] Liang G, Chan MF, Tomigahara Y, Tsai YC, Gonzales FA (2002). Cooperativity between DNA methyltransferases in the maintenance methylation of repetitive elements. Mol Cell Biol.

[71] Chen T, Ueda Y, Dodge JE, Wang Z, Li E (2003). Establishment and maintenance of genomic methylation patterns in mouse embryonic stem cells by Dnmt3a and Dnmt3b. Mol Cell Biol.

[72] Jeong S, Liang G, Sharma S, Lin JC, Choi SH (2009). Selective anchoring of DNA methyltransferases 3A and 3B to nucleosomes containing methylated DNA. Mol Cell Biol.

[73] Ting AH, Jair KW, Suzuki H, Yen RW, Baylin SB (2004). CpG island hypermethylation is maintained in human colorectal cancer cells after RNAi-mediated depletion of DNMT1. Nat Genet.

[74] Rhee I, Bachman KE, Park BH, Jair KW, Yen RW (2002). DNMT1 and DNMT3b cooperate to silence genes in human cancer cells. Nature.

[75] Datta J, Majumder S, Bai S, Ghoshal K, Kutay H (2005). Physical and functional interaction of DNA methyltransferase 3A with Mbd3 and Brg1 in mouse lymphosarcoma cells. Cancer Res.

[76] Hervouet E, Vallette FM, Cartron PF (2010). Impact of the DNA methyltransferases expression on the methylation status of apoptosis-associated genes in glioblastoma multiforme. Cell Death Dis.

[77] Gowher H, Jeltsch A (2002). Molecular enzymology of the catalytic domains of the Dnmt3a and Dnmt3b DNA methyltransferases. J Biol Chem.

[78] Geiman TM, Sankpal UT, Robertson AK, Chen Y, Mazumdar M (2004). Isolation and characterization of a novel DNA methyltransferase complex linking DNMT3B with components of the mitotic chromosome condensation machinery. Nucleic Acids Res.

[79] Margot JB, Cardoso MC, Leonhardt H (2001). Mammalian DNA methyltransferases show different subnuclear distributions. J Cell Biochem.

[80] Shamay M, Greenway M, Liao G, Ambinder RF, Hayward SD (2010). De novo DNA methyltransferase DNMT3b interacts with NEDD8-modified proteins. J Biol Chem.

[81] Hu JL, Zhou BO, Zhang RR, Zhang KL, Zhou JQ (2009). The N-terminus of histone H3 is required for de novo DNA methylation in chromatin. Proc Natl Acad Sci U S A.

[82] Jia D, Jurkowska RZ, Zhang X, Jeltsch A, Cheng X (2007). Structure of Dnmt3a bound to Dnmt3L suggests a model for de novo DNA methylation. Nature.

[83] Ooi SK, Qiu C, Bernstein E, Li K, Jia D (2007). DNMT3L connects unmethylated lysine 4 of histone H3 to de novo methylation of DNA. Nature.

[84] Zhang Y, Jurkowska R, Soeroes S, Rajavelu A, Dhayalan A (2010). Chromatin methylation activity of Dnmt3a and Dnmt3a/3L is guided by interaction of the ADD domain with the histone H3 tail. Nucleic Acids Res.

[85] Otani J, Nankumo T, Arita K, Inamoto S, Ariyoshi M (2009). Structural basis for recognition of H3K4 methylation status by the DNA methyltransferase 3A ATRX-DNMT3-DNMT3L domain. EMBO Rep.

[86] Kareta MS, Botello ZM, Ennis JJ, Chou C, Chedin F (2006). Reconstitution and mechanism of the stimulation of de novo methylation by human DNMT3L. J Biol Chem.

[87] Suetake I, Shinozaki F, Miyagawa J, Takeshima H, Tajima S (2004). DNMT3L stimulates the DNA methylation activity of Dnmt3a and Dnmt3b through a direct interaction. J Biol Chem.

[88] Chen ZX, Mann JR, Hsieh CL, Riggs AD, Chedin F (2005). Physical and functional interactions between the human DNMT3L protein and members of the de novo methyltransferase family. J Cell Biochem.

[89] Glass JL, Fazzari MJ, Ferguson-Smith AC, Greally JM (2009). CG dinucleotide periodicities recognized by the Dnmt3a-Dnmt3L complex are distinctive at retroelements and imprinted domains. Mamm Genome.

[90] Kaneda M, Okano M, Hata K, Sado T, Tsujimoto N (2004). Essential role for de novo DNA methyltransferase Dnmt3a in paternal and maternal imprinting. Nature.

[91] Hata K, Okano M, Lei H, Li E (2002). Dnmt3L cooperates with the Dnmt3 family of de novo DNA methyltransferases to establish maternal imprints in mice. Development.

[92] Nimura K, Ishida C, Koriyama H, Hata K, Yamanaka S (2006). Dnmt3a2 targets endogenous Dnmt3L to ES cell chromatin and induces regional DNA methylation. Genes Cells.

[93] Feldman N, Gerson A, Fang J, Li E, Zhang Y (2006). G9a-mediated irreversible epigenetic inactivation of Oct-3/4 during early embryogenesis. Nat Cell Biol.

[94] Zhu H, Geiman TM, Xi S, Jiang Q, Schmidtmann A (2006). Lsh is involved in de novo methylation of DNA. EMBO J.

[95] Xi S, Geiman TM, Briones V, Guang Tao Y, Xu H (2009). Lsh participates in DNA methylation and silencing of stem cell genes. Stem Cells.

[96] Fan T, Schmidtmann A, Xi S, Briones V, Zhu H (2008). DNA hypomethylation caused by Lsh deletion promotes erythroleukemia development. Epigenetics.

[97] Myant K, Stancheva I (2008). LSH cooperates with DNA methyltransferases to repress transcription. Mol Cell Biol.

[98] Deplus R, Brenner C, Burgers WA, Putmans P, Kouzarides T (2002). Dnmt3L is a transcriptional repressor that recruits histone deacetylase. Nucleic Acids Res.

[99] Aapola U, Liiv I, Peterson P (2002). Imprinting regulator DNMT3L is a transcriptional repressor associated with histone deacetylase activity. Nucleic Acids Res.

[100] Datta J, Ghoshal K, Sharma SM, Tajima S, Jacob ST (2003). Biochemical fractionation reveals association of DNA methyltransferase (Dnmt) 3b with Dnmt1 and that of Dnmt 3a with a histone H3 methyltransferase and Hdac1. J Cell Biochem.

[101] Fuks F, Burgers WA, Godin N, Kasai M, Kouzarides T (2001). Dnmt3a binds deacetylases and is recruited by a sequence-specific repressor to silence transcription. EMBO J.

[102] Geiman TM, Sankpal UT, Robertson AK, Zhao Y, Robertson KD (2004). DNMT3B interacts with hSNF2H chromatin remodeling enzyme, HDACs 1 and 2, and components of the histone methylation system. Biochem Biophys Res Commun.

[103] Zhao Q, Rank G, Tan YT, Li H, Moritz RL (2009). PRMT5-mediated methylation of histone H4R3 recruits DNMT3A, coupling histone and DNA methylation in gene silencing. Nat Struct Mol Biol.

[104] Dhayalan A, Rajavelu A, Rathert P, Tamas R, Jurkowska RZ (2010). The Dnmt3a PWWP domain reads histone 3 lysine 36 trimethylation and guides DNA methylation. J Biol Chem.

[105] Gopalakrishnan S, Sullivan BA, Trazzi S, Della Valle G, Robertson KD (2009). DNMT3B interacts with constitutive centromere protein CENP-C to modulate DNA methylation and the histone code at centromeric regions. Hum Mol Genet.

[106] El Gazzar M, Yoza BK, Chen X, Hu J, Hawkins GA (2008). G9a and HP1 couple histone and DNA methylation to TNFalpha transcription silencing during endotoxin tolerance. J Biol Chem.

[107] Li H, Rauch T, Chen ZX, Szabo PE, Riggs AD (2006). The histone methyltransferase SETDB1 and the DNA methyltransferase DNMT3A interact directly and localize to promoters silenced in cancer cells. J Biol Chem.

[108] Han P, Li W, Yang J, Shang C, Lin CH (2016). Epigenetic response to environmental stress: assembly of BRG1-G9a/GLP-DNMT3 repressive chromatin complex on Myh6 promoter in pathologically stressed hearts. Biochim Biophys Acta.

[109] Chang Y, Sun L, Kokura K, Horton JR, Fukuda M (2011). MPP8 mediates the interactions between DNA methyltransferase Dnmt3a and H3K9 methyltransferase GLP/G9a. Nat Commun.

[110] Meilinger D, Fellinger K, Bultmann S, Rothbauer U, Bonapace IM (2009). Np95 interacts with de novo DNA methyltransferases, Dnmt3a and Dnmt3b, and mediates epigenetic silencing of the viral CMV promoter in embryonic stem cells. EMBO Rep.

[111] Fatemi M, Hermann A, Gowher H, Jeltsch A (2002). Dnmt3a and Dnmt1 functionally cooperate during de novo methylation of DNA. Eur J Biochem.

[112] Jair KW, Bachman KE, Suzuki H, Ting AH, Rhee I (2006). De novo CpG island methylation in human cancer cells. Cancer Res.

[113] Egger G, Jeong S, Escobar SG, Cortez CC, Li TW (2006). Identification of DNMT1 (DNA methyltransferase 1) hypomorphs in somatic knockouts suggests an essential role for DNMT1 in cell survival. Proc Natl Acad Sci U S A.

[114] Fatemi M, Hermann A, Pradhan S, Jeltsch A (2001). The activity of the murine DNA methyltransferase Dnmt1 is controlled by interaction of the catalytic domain with the N-terminal part of the enzyme leading to an allosteric activation of the enzyme after binding to methylated DNA. J Mol Biol.

[115] Felle M, Joppien S, Nemeth A, Diermeier S, Thalhammer V (2011). The USP7/Dnmt1 complex stimulates the DNA methylation activity of Dnmt1 and regulates the stability of UHRF1. Nucleic Acids Res.

[116] Foltz G, Yoon JG, Lee H, Ryken TC, Sibenaller Z (2009). DNA methyltransferase-mediated transcriptional silencing in malignant glioma: a combined whole-genome microarray and promoter array analysis. Oncogene.

[117] Lin IG, Han L, Taghva A, O'Brien LE, Hsieh CL (2002). Murine de novo methyltransferase Dnmt3a demonstrates strand asymmetry and site preference in the methylation of DNA in vitro. Mol Cell Biol.

[118] Takahashi M, Kamei Y, Ehara T, Yuan X, Suganami T (2013). Analysis of DNA methylation change induced by Dnmt3b in mouse hepatocytes. Biochem Biophys Res Commun.

[119] Mortusewicz O, Schermelleh L, Walter J, Cardoso MC, Leonhardt H (2005). Recruitment of DNA methyltransferase I to DNA repair sites. Proc Natl Acad Sci U S A.

[120] Guo G, Wang W, Bradley A (2004). Mismatch repair genes identified using genetic screens in Blm-deficient embryonic stem cells. Nature.

[121] Yu Z, Kong Q, Kone BC (2013). Aldosterone reprograms promoter methylation to regulate alphaENaC transcription in the collecting duct. Am J Physiol Renal Physiol.

[122] Boland MJ, Christman JK (2008). Characterization of Dnmt3b:thymine-DNA glycosylase interaction and stimulation of thymine glycosylase-mediated repair by DNA methyltransferase(s) and RNA. J Mol Biol.

[123] Li YQ, Zhou PZ, Zheng XD, Walsh CP, Xu GL (2007). Association of Dnmt3a and thymine DNA glycosylase links DNA methylation with base-excision repair. Nucleic Acids Res.

[124] O'Hagan HM, Mohammad HP, Baylin SB (2008). Double strand breaks can initiate gene silencing and SIRT1-dependent onset of DNA methylation in an exogenous promoter CpG island. PLoS Genet.

[125] Metivier R, Gallais R, Tiffoche C, Le Peron C, Jurkowska RZ (2008). Cyclical DNA methylation of a transcriptionally active promoter. Nature.

[126] Gazin C, Wajapeyee N, Gobeil S, Virbasius CM, Green MR (2007). An elaborate pathway required for Ras-mediated epigenetic silencing. Nature.

[127] Negishi M, Saraya A, Miyagi S, Nagao K, Inagaki Y (2007). Bmi1 cooperates with Dnmt1-associated protein 1 in gene silencing. Biochem Biophys Res Commun.

[128] Yamashita M, Kuwahara M, Suzuki A, Hirahara K, Shinnaksu R (2008). Bmi1 regulates memory CD4 T cell survival via repression of the Noxa gene. J Exp Med.

[129] Wu X, Gong Y, Yue J, Qiang B, Yuan J (2008). Cooperation between EZH2, NSPc1-mediated histone H2A ubiquitination and Dnmt1 in HOX gene silencing. Nucleic Acids Res.

[130] Vire E, Brenner C, Deplus R, Blanchon L, Fraga M (2006). The polycomb group protein EZH2 directly controls DNA methylation. Nature.

[131] Jin B, Yao B, Li JL, Fields CR, Delmas AL (2009). DNMT1 and DNMT3B modulate distinct polycomb-mediated histone modifications in colon cancer. Cancer Res.

[132] Rush M, Appanah R, Lee S, Lam LL, Goyal P (2009). Targeting of EZH2 to a defined genomic site is sufficient for recruitment of Dnmt3a but not de novo DNA methylation. Epigenetics.

[133] Athanasiadou R, de Sousa D, Myant K, Merusi C, Stancheva I (2010). Targeting of de novo DNA methylation throughout the Oct-4 gene regulatory region in differentiating embryonic stem cells. PLoS One.

[134] Gu P, Xu X, Le Menuet D, Chung AC, Cooney AJ (2011). Differential recruitment of methyl CpG-binding domain factors and DNA methyltransferases by the orphan receptor germ cell nuclear factor initiates the repression and silencing of Oct4. Stem Cells.

[135] Epsztejn-Litman S, Feldman N, Abu-Remaileh M, Shufaro Y, Gerson A (2008). De novo DNA methylation promoted by G9a prevents reprogramming of embryonically silenced genes. Nat Struct Mol Biol.

[136] Zheng DL, Zhang L, Cheng N, Xu X, Deng Q (2009). Epigenetic modification induced by hepatitis B virus X protein via interaction with de novo DNA methyltransferase DNMT3A. J Hepatol.

[137] Park IY, Sohn BH, Yu E, Suh DJ, Chung YH (2007). Aberrant epigenetic modifications in hepatocarcinogenesis induced by hepatitis B virus X protein. Gastroenterology.

[138] Zhou Y, Grummt I (2005). The PHD finger/bromodomain of NoRC interacts with acetylated histone H4K16 and is sufficient for rDNA silencing. Curr Biol.

[139] Majumder S, Ghoshal K, Datta J, Smith DS, Bai S (2006). Role of DNA methyltransferases in regulation of human ribosomal RNA gene transcription. J Biol Chem.

[140] Hervouet E, Vallette FM, Cartron PF (2010). Dnmt1/transcription factor interactions: an alternative mechanism of DNA methylation inheritance. Genes Cancer.

[141] Hervouet E, Nadaradjane A, Gueguen M, Vallette FM, Cartron PF (2012). Kinetics of DNA methylation inheritance by the Dnmt1-including complexes during the cell cycle. Cell Div.

[142] Esteve PO, Chin HG, Pradhan S (2005). Human maintenance DNA (cytosine-5)-methyltransferase and p53 modulate expression of p53-repressed promoters. Proc Natl Acad Sci U S A.

[143] Hayashi N, Kobayashi M, Shamma A, Morimura Y, Takahashi C (2013). Regulatory interaction between NBS1 and DNMT1 responding to DNA damage.

[144] Le Gac G, Esteve PO, Ferec C, Pradhan S (2006). DNA damage-induced down-regulation of human Cdc25C and Cdc2 is mediated by cooperation between p53 and maintenance DNA (cytosine-5) methyltransferase 1. J Biol Chem.

[145] Arabsolghar R, Azimi T, Rasti M. Mutant p53 binds to estrogen receptor negative promoter via DNMT1 and HDAC1 in MDA-MB-468 breast cancer cells. Mol Biol Rep. 2013;40(3):2617-25. 10.1007/s11033-012-2348-7 23242655

[146] Liu S, Shen T, Huynh L, Klisovic MI, Rush LJ, et al. Interplay of RUNX1/MTG8 and DNA methyltransferase 1 in acute myeloid leukemia. Cancer Res. 2005;65(4):1277-84. 10.1158/0008-5472.CAN-04-4532 15735013

[147] Sajedi E, Gaston-Massuet C, Andoniadou CL, Signore M, Hurd PJ (2008). DNMT1 interacts with the developmental transcriptional repressor HESX1. Biochim Biophys Acta.

[148] Zhang Q, Wang HY, Marzec M, Raghunath PN, Nagasawa T (2005). STAT3- and DNA methyltransferase 1-mediated epigenetic silencing of SHP-1 tyrosine phosphatase tumor suppressor gene in malignant T lymphocytes. Proc Natl Acad Sci U S A.

[149] Puto LA, Reed JC (2008). Daxx represses RelB target promoters via DNA methyltransferase recruitment and DNA hypermethylation. Genes Dev.

[150] Zhang H, He J, Li J, Tian D, Gu L (2013). Methylation of RASSF1A gene promoter is regulated by p53 and DAXX. FASEB J.

[151] Wang YA, Kamarova Y, Shen KC, Jiang Z, Hahn MJ (2005). DNA methyltransferase-3a interacts with p53 and represses p53-mediated gene expression. Cancer Biol Ther.

[152] Suzuki M, Yamada T, Kihara-Negishi F, Sakurai T, Hara E (2006). Site-specific DNA methylation by a complex of PU.1 and Dnmt3a/b. Oncogene.

[153] Senyuk V, Premanand K, Xu P, Qian Z, Nucifora G (2011). The oncoprotein EVI1 and the DNA methyltransferase Dnmt3 co-operate in binding and de novo methylation of target DNA. PLoS One.

[154] Cheray M, Pacaud R, Nadaradjane A, Oliver L, Vallette FM (2016). Specific inhibition of DNMT3A/ISGF3gamma interaction increases the temozolomide efficiency to reduce tumor growth. Theranostics.

[155] Brenner C, Deplus R, Didelot C, Loriot A, Vire E (2005). Myc represses transcription through recruitment of DNA methyltransferase corepressor. EMBO J.

[156] Hervouet E, Vallette FM, Cartron PF (2009). Dnmt3/transcription factor interactions as crucial players in targeted DNA methylation. Epigenetics.

[157] Zhang J, Zhou C, Jiang H, Liang L, Shi W (2017). ZEB1 induces ER-alpha promoter hypermethylation and confers antiestrogen resistance in breast cancer. Cell Death Dis.

[158] Zhou C, Jiang H, Zhang Z, Zhang G, Wang H (2017). ZEB1 confers stem cell-like properties in breast cancer by targeting neurogenin-3. Oncotarget.

[159] Di Croce L, Raker VA, Corsaro M, Fazi F, Fanelli M (2002). Methyltransferase recruitment and DNA hypermethylation of target promoters by an oncogenic transcription factor. Science.

[160] Velasco G, Hube F, Rollin J, Neuillet D, Philippe C (2010). Dnmt3b recruitment through E2F6 transcriptional repressor mediates germ-line gene silencing in murine somatic tissues. Proc Natl Acad Sci U S A.

[161] Robertson KD, Ait-Si-Ali S, Yokochi T, Wade PA, Jones PL (2000). DNMT1 forms a complex with Rb, E2F1 and HDAC1 and represses transcription from E2F-responsive promoters. Nat Genet.

[162] Shamay M, Krithivas A, Zhang J, Hayward SD (2006). Recruitment of the de novo DNA methyltransferase Dnmt3a by Kaposi's sarcoma-associated herpesvirus LANA. Proc Natl Acad Sci U S A.

[163] Pacaud R, Sery Q, Oliver L, Vallette FM, Tost J (2014). DNMT3L interacts with transcription factors to target DNMT3L/DNMT3B to specific DNA sequences: role of the DNMT3L/DNMT3B/p65-NFkappaB complex in the (de-)methylation of TRAF1. Biochimie.

[164] Cartron PF, Blanquart C, Hervouet E, Gregoire M, Vallette FM. HDAC1-mSin3a-NCOR1, Dnmt3b-HDAC1-Egr1 and Dnmt1-PCNA-UHRF1-G9a regulate the NY-ESO1 gene expression. Mol Oncol. 2012; 10.1016/j.molonc.2012.11.004 PMC5528493 23312906

